# Case Report: A prenatal diagnosis of osteogenesis imperfecta in a patient with a novel pathogenic variant in *COL1A2*

**DOI:** 10.12688/f1000research.131094.1

**Published:** 2023-06-05

**Authors:** Melissa Sindy Peláez Chomba, Guillermo Raúl Vásquez Gómez, Yasser Ciro Sullcahuaman Allende, Julio Cesar Mendoza Fernández, Nelson David Purizaca Rosillo, Alejandra Zevallos, Vicente Leandro Cruzate Cabrejos

**Affiliations:** 1 Departamento de Gineco Obstetricia, Hospital Nacional Docente Madre Niño San Bartolomé, Lima, Lima, Cercado de Lima 15001, Peru; 2 Instituto de Investigación Genómica, IGENOMICA, Lima, Lima, San Borja 15037, Peru; 3 School of Medicine, Universidad Peruana Cayetano Heredia, Lima, Lima, San Martin de Porres 15102, Peru; 4 Unidad Funcional de Genética y Biología Molecular, Instituto Nacional de Enfermedades Neoplásicas, Lima, Lima, Surquillo 15038, Peru; 5 Escuela Profesional de Medicina Humana, Universidad Privada San Juan Bautista, Ica, Ica, Subtanjalla 11004, Peru; 6 Escuela Profesional de Medicina Humana, Universidad Privada San Juan Bautista, Lima, Lima, Chorrillos 15067, Peru

**Keywords:** Osteogenesis imperfecta, newborn, prenatal diagnosis

## Abstract

Osteogenesis imperfecta is considered a rare genetic condition which is characterized by bone fragility. In 85% of cases, it is caused by mutations in *COL1A1* and *COL1A2* genes which are essential to produce type I collagen. We report the case of a female neonate delivered to a 27-year-old women at San Bartolomé Teaching Hospital with a family history of clavicle fracture. A prenatal control with ultrasound was performed to the mother at 29 weeks. A fetus with altered morphology and multiple fractures was found. Therefore, a prenatal diagnosis of osteogenesis imperfecta was performed. The neonate was born with a respiratory distress syndrome and an acyanotic congenital heart disease. Therefore, she remained in NICU until her death. We highlight the importance of prenatal diagnosis, genetic counseling and a multidisciplinary evaluation in this type of pathologies and report a new probably pathogenic variant in the *COL1A2* gene detected by exomic sequencing in amniotic fluid.

## Introduction

Skeletal disorders (SD) are a genetically diverse group of 461 different diseases classified into 42 groups. ^1^ This group of rare bone diseases accounts for 5% of all birth defects. ^2^ Among the most common SD in the prenatal stage are achondroplasia, thanatophoric dysplasia, osteogenesis imperfecta (OI), and achondrogenesis. ^3^

OI is a connective tissue genetic disorder with an incidence of 1 in every 15 to 20,000 births and comprises a heterogeneous group of diseases characterized by susceptibility to bone fractures of varying severity. ^4^ The clinical picture presents a wide intra- and interfamily variability, which ranges from individuals with occasional fractures and regular height, to phenotypes characterized by severe skeletal fragility, bone deformities, and significant growth deficiency, to neonatal lethality. Other signs include blue sclera, hearing loss, dentinogenesis imperfecta, heart defects, and joint hypermobility. ^4^^,^^5^

The classification of OI has undergone significant changes with the discovery of new genes, and there is great genetic heterogeneity, including forms of autosomal dominant, autosomal recessive, and X-linked inheritance. Currently, five (05) types of OI are considered, of which around 90% of cases are associated with heterozygous pathogenic/probable pathogenic (PV/PVP) variants in the *COL1A1* or *COL1A2* genes. ^1^^,^^6^

The objective of the present case was to report the prenatal diagnosis of osteogenesis imperfecta associated with a new probably pathogenic variant in the *COL1A2* gene detected by exomic sequencing in amniotic fluid.

## Methods

For the extraction of fetal DNA, amniocentesis was performed at 29 weeks and 4 days of gestation, collecting 3 ml of amniotic fluid in an EDTA tube. The sample was labeled and processed by centrifugation at 3000 rpm for 10 minutes to obtain the cell pellet. After washing with Phosphate Buffered Saline (PBS) pH 7.4, the cells resuspended in the same buffer were aliquoted. DNA extraction was performed with the ROCHE® High Pure PCR Template Preparation Kit according to the manufacturer's instructions. The eluate (30uL) was quantified using an Invitrogen Qubit 4 fluorometer and stored at -20°C until use.

For complete exome sequencing using massive sequencing by synthesis (NGS-Exome) technology, the sample was sent by air to Macrogen according to the requested requirements: Sample [58.348ng/uL] with genomic DNA integrity test by electrophoresis in agarose. The platform used was the following. Sequencing on Novaseq 6000, 150bp PE. Library preparation: Exome capture (SureSelect V6 post capture kit).

## Case report

**Figure 1.**
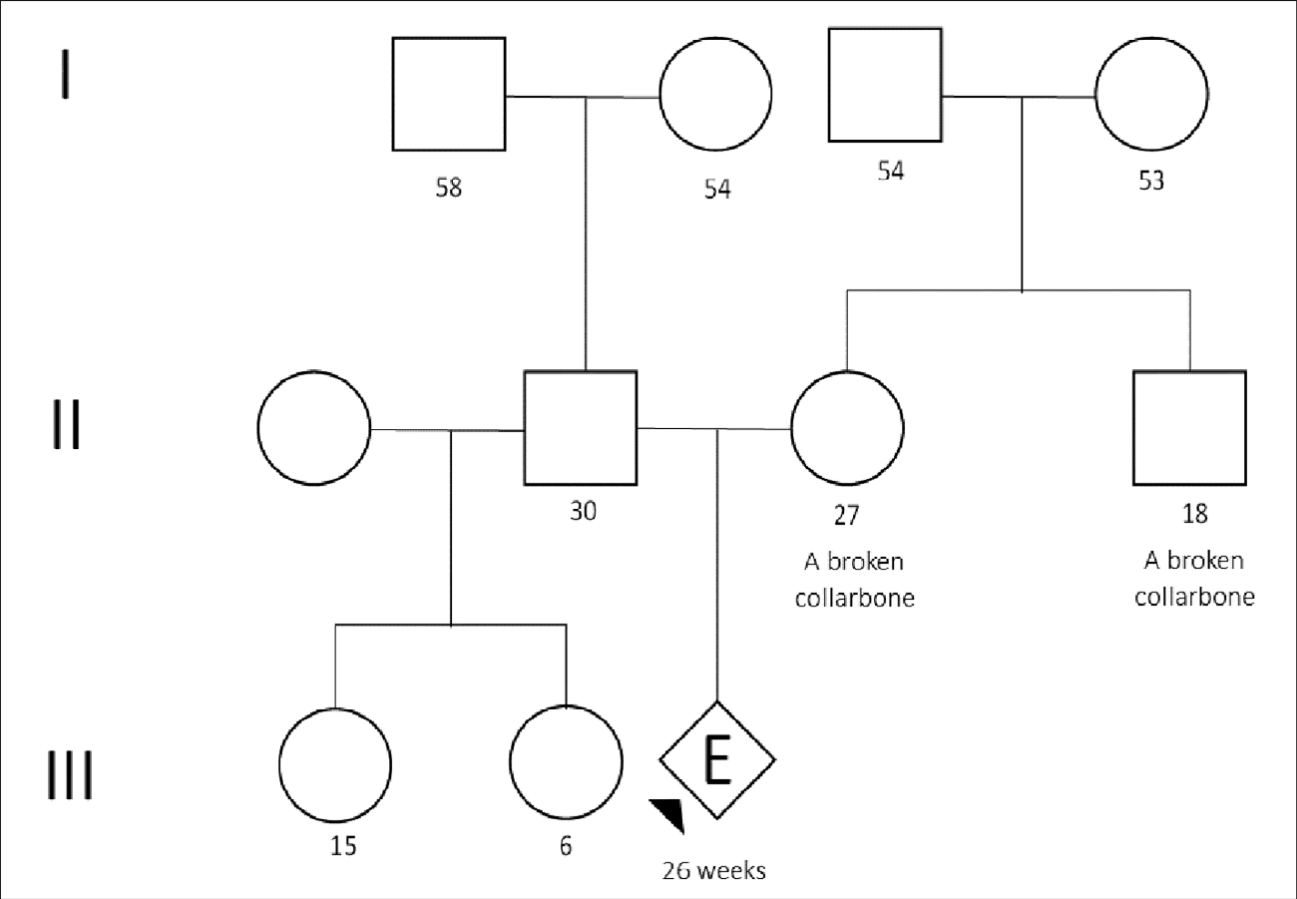
Pedigree (arrows indicate the persons tested).

The proband was the first child of a 27-year-old Peruvian mother who works as a call center agent. The mother had a personal and family history of clavicle fracture ( Figure 1). An ultrasound was performed in another institution at 22 weeks, which suggested a clinical suspicion of achondroplasia. Therefore, she was referred to the Saint Bartolome National Hospital for specialized treatment.

**Figure 2.**
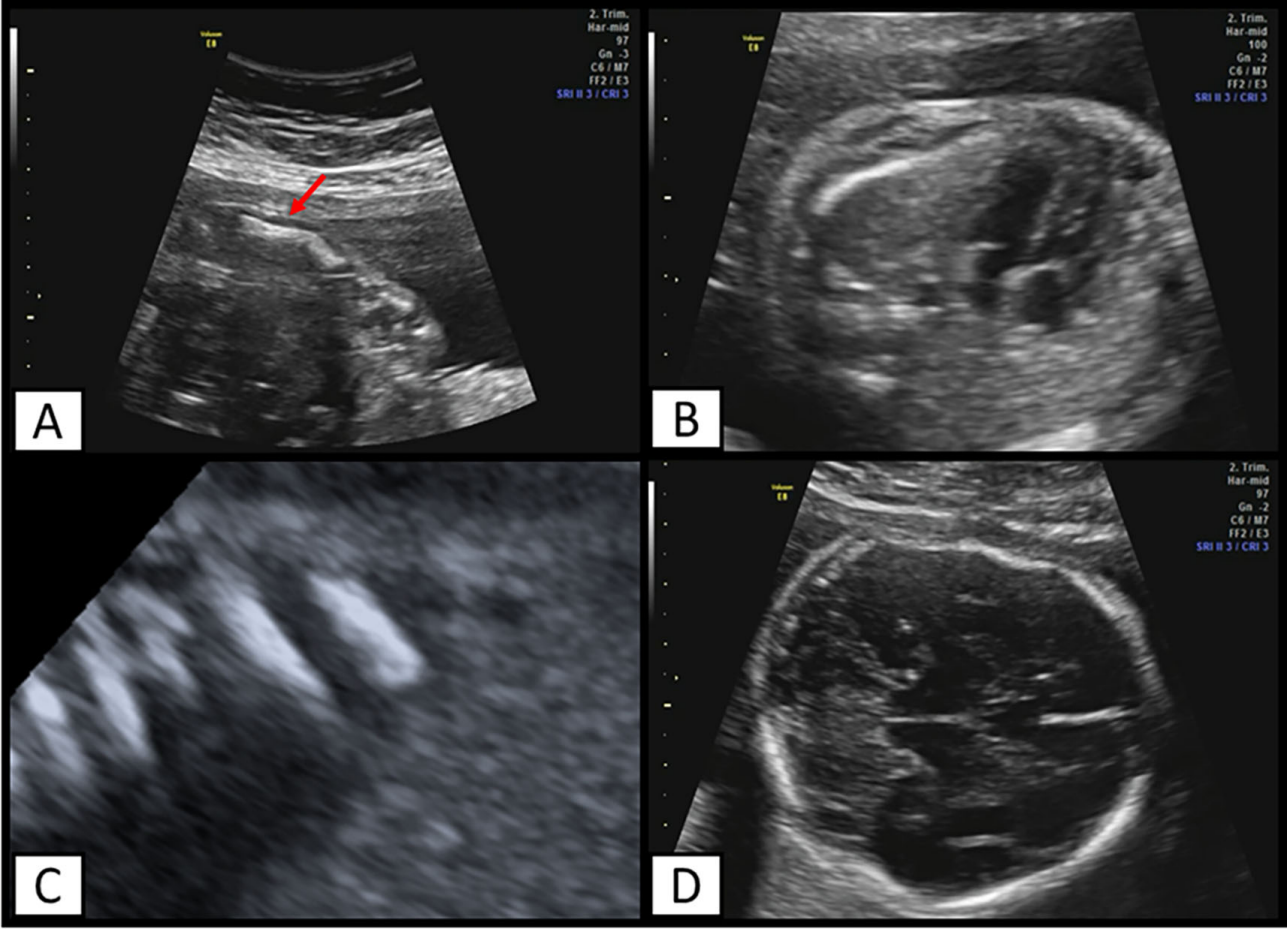
Ultrasound findings. A) Ulna with altered morphology. Arrow indicates a point fracture, B) Ribs that exceed the middle of the thorax, C) multiple rib fractures. D) Deformation of fetal head and cranial fractures can also be observed.

The mother was admitted to our institution at 23 weeks and 2 days, an ultrasound evaluation was performed, finding a podalic fetus with reduced mobility, long bones below the fifth percentile (corresponding to biometry at 16 weeks), altered morphology ( Figure 2A), and continuity solution, in addition to femur length/abdominal circumference=0.11. Fractures were also found at the level of the femur, tibia, fibula, ulna, and ribs ( Figure 2B, C) and at the cephalic level there was a skull deformity on pressure echocardiography and cranial fractures ( Figure 2D).

In the thoracic evaluation, the ribs comprise two thirds of the fetal thorax, suggesting a low risk of pulmonary hypoplasia. Finally, it was concluded that she was pregnant at 21.3 weeks by fetal biometry, with a probable skeletal disorder, for which an evaluation by the Genetics Office was requested.

After providing genetic counseling and a multidisciplinary evaluation, a complete exome sequencing study was proposed in amniotic fluid and with parental consent before amniocentesis was performed at 29 weeks and 4 days.

The variant c.1612G>T (p.Gly538Cys) was identified in the *COL1A2* gene (NM_000089.4) in a heterozygous state. This variant was evaluated according to the criteria recommended by the American College of Medical Genetics and Genomics (ACMG) and classified as a likely pathogenic variant. ^7^ The clinical findings of the patient and the presence of the probably pathogenic variant in the *COL1A2* gene confirmed the diagnosis of Osteogenesis Imperfecta type II.

**Figure 3.**
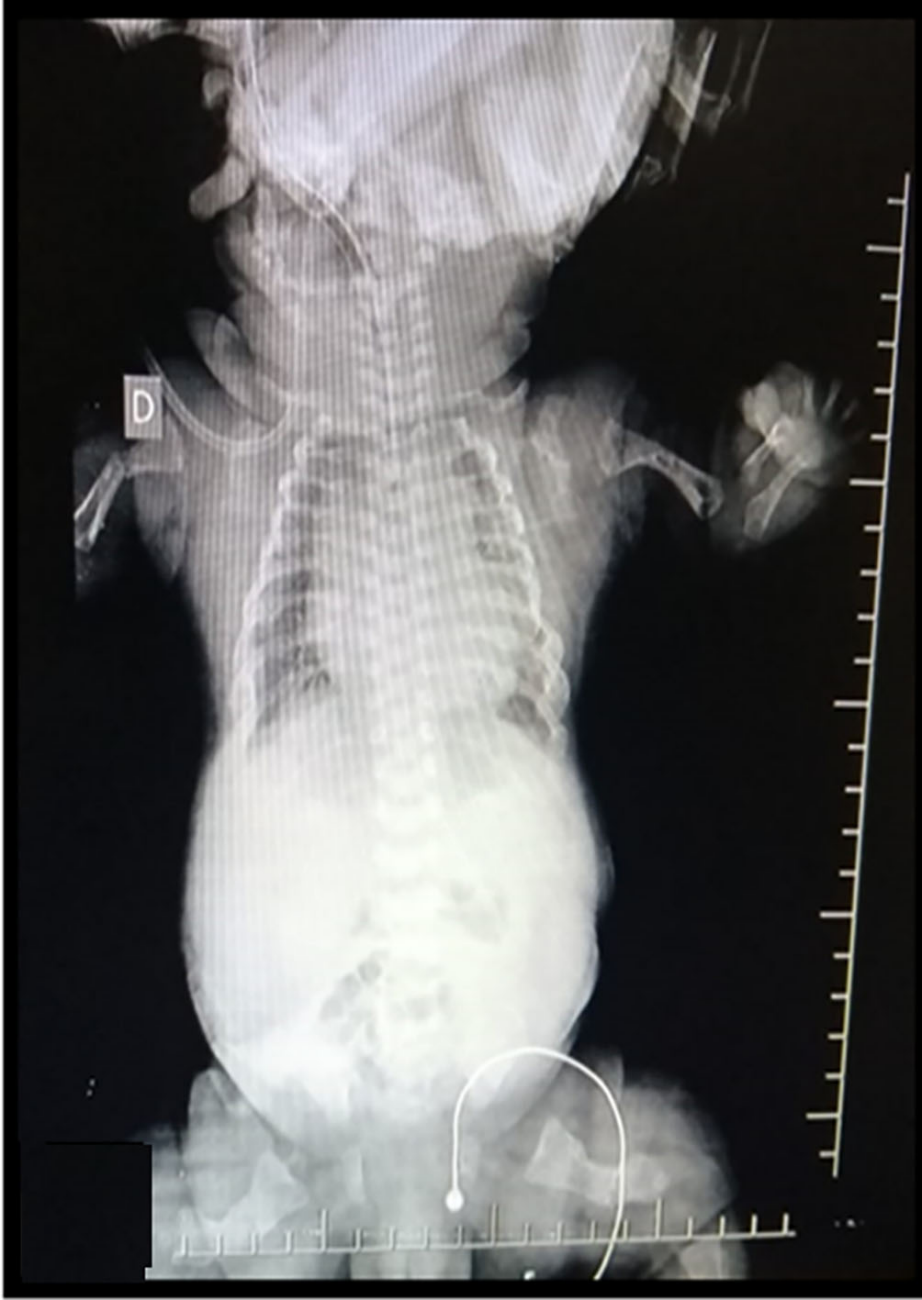
Radiographs from proband which presented hypomineralization and lack of growth of the long bones.

After 9 prenatal controls in the institution, an elective cesarean section was scheduled at 38 weeks and 6 days, delivering a female newborn with a weighi of 3.330 kg, height 43cm, head circumference 34.5cm, chest circumference 34cm, APGAR 5 at minute, 6 at 5 minutes, and 7 at 10 minutes, associated with respiratory distress, which required endotracheal intubation and hospitalization in the neonatal intensive care unit (NICU) ( Figure 3). She was admitted with diagnoses of a 39-week-old newborn, osteogenesis imperfecta type II, and respiratory distress syndrome to rule out acyanotic congenital heart disease.

Doppler echocardiography reported a bicuspid aortic valve, severe aortic stenosis, plus hypoplasia of the aortic ring without a significant obstructive gradient; as well as a cardiothoracic index of 0.70, which is considered pulmonary hypoplasia.

Advanced life support was provided to the newborn for cardiogenic shock with vasoactive drugs to improve pre- and post-loading. The drugs used were norepinephrine and dobutamine in continuous infusion at low doses. Additionally, a drug treatment with furosemide and captopril at low doses were administrated to control congestive heart failure.

On the other hand, Intratracheal intubation was performed following initial sedoanalgesia protocols with 0.2mg/kg midazolam. Subsequently, she was maintained with sedoanalgesia and 0.1mg/kg morphine for pain management and dyspnea. She remained hospitalized for 17 days in the NICU with an invasive ventilation support to improve gas exchange and mechanical ventilation. An Enteral nutrition was given with calcium and vitamin D and a prophylactic iron dose of 2mg/kg/day. Furthermore, Anasarca was reduced with diuretic and colloid therapy.

On mechanical ventilation, the newborn developed pneumonia and was treated with antimicrobial agents for 7 days based on the culture results (Ampicillin: 50 mg/kg every 8 h and Gentamicin: 3 mg/kg once a day). Finally, despite intermittent episodes of dyspnea, the patient's progression was stable, therefore, she was referred to the neonatal unit for follow-up care. However, six days after being discharged from NICU, she was readmitted for a sudden worsening and died from a cardiopulmonary arrest.

## Discussion

OI is a genetic disorder of connective tissue with an incidence of 1 in every 15 to 20,000 births. It comprises a heterogeneous group of diseases, with autosomal dominant, autosomal recessive, and X-linked inheritance patterns, whose clinical picture is primarily characterised by susceptibility to bone fractures to a variable degree, as well as bone deformities, significant growth deficiency, blue sclera, hearing loss, dentinogenesis imperfecta, heart defects, and joint hypermobility. ^4^^,^^5^^,^^8^

Classically, the classification of OI included up to 18 types, ^9^ which have undergone significant changes with the discovery of new genes. Currently, IAO is part of the 25 ED groups, called the Osteogenesis Imperfecta and Decreased Bone Density Group, and five types of IAO are considered, ^1^^,^^6^ of which approximately 90% of cases are associated with heterozygous PV/PVP in the *COL1A1* or *COL1A2* genes. VP/VPP in the *COL1A1* or *COL1A2* genes alter the alpha-1 and alpha-2 chains of type I collagen, respectively, which is the main component of the bone matrix, generating alterations in the bone architecture that explain the clinical manifestations of OI. ^8^^,^^9^

Regarding the prenatal parameters found in the patient, chest circumference <p5 for gestational age, chest circumference <0.75 and relative cardiomegaly have a sensitivity of 55-80%, a specificity of 90-100%, a positive predictive value of 80-100%, and a negative predictive value of 87-91% for the association with pulmonary hypoplasia. The characteristics found in the prenatal ultrasound evaluation, such as altered morphology of the long bones, foot malposition, rib, and skull fractures, support the clinical diagnosis of Osteogenesis Imperfecta. ^4^^,^^5^^,^^8^

Postnatal physical examination revealed height below the third percentile, blue sclera, short nose, and micromelia. Both thighs were abducted and in external rotation. Neonatal radiography confirmed the presence of curvature and humeral and femur fractures, as well as rib fractures and generalized hypomineralization. Finally, the patient died at 23 days. These findings coincide with the phenotype of OI type II and the reports that 90% of these patients die at 4 weeks of age. ^4^^,^^5^^,^^8^

There are several ways to diagnose type II OI. The mainstay is prenatal ultrasound, which can reveal bone fractures during pregnancy. Skeletal abnormalities can be detected as early as 14 weeks, regardless of whether there is a family history. ^10^ It is important to note that misdiagnosis of the foetus during a prenatal ultrasound is common, as ultrasound details might be unreliable. ^11^^,^^12^ Therefore, biochemical analysis, such as analysis of collagen production in fibroblast cultures or DNA extraction from blood to verified mutations, is important. ^13^^,^^14^ However, technologies such as next-generation sequencing have led to a better personalization of medical care in pathologies such as OI, as they enable a faster diagnosis and a more accurate identification of the polymorphisms. ^15^ Moreover, an accurate diagnosis has a direct impact on the selection of patient treatment. On the other hand, a prenatal diagnosis improves the genetic counseling offered to parents, since it is common that two healthy parents have a child with OI and determine whether it is a sporadic mutation or inherited could be crucial for effective case-to-case surveillance. ^16^^,^^17^

In the present case, the patient's prenatal clinical diagnosis was made through an exome sequencing study with amniotic fluid. We identified the variant c.1612G>T (p.Gly538Cys) in the *COL1A2* gene (NM_000089.4) in a heterozygous state. Most cases of type II-IV OI are associated with heterozygous variants in the *COL1A1* and *COL1A2* genes that generate glycine substitutions. About 80% of these variants have been reported to be of missense type, and, in general, glycine substitutions near the carboxyl-terminus are associated with a more severe phenotype. ^8^^,^^18^ We must highlight that this variant is not registered with gnomAD or CLINVAR. Likewise, it is found in exon 28, one base away from the splicing site; furthermore, different computational predictors (REVEL, SIFT, PROVEAN, MutPred, DEOGEN2, MutationTaster) report it as harmful or pathogenic. Based on the criteria recommended by ACMG, this variant was classified as probably pathogenic. ^7^ With these clinical and molecular prenatal findings, the diagnosis of osteogenesis Imperfecta type II was confirmed as associated with a probable pathogenic variant in the *COL1A2* gene, also known as the lethal perinatal form. ^1^

Currently, exome sequencing is a tool that allows for better clinical management of complex cases. According to a study by Jenny Lord *et al.* ^19^ reported that the application of exome sequencing facilitates the genetic diagnosis of ultrasound-detected fetal structural abnormalities, allowing the prediction of the prognosis and the risk of recurrence in future pregnancies, but the use of exome sequencing should be carefully considered. case selection to maximize clinical utility. Similarly, Zhao *et al.* ^20^ report that the clinical application of exome sequencing allows detecting the monogenic etiology of pregnancy loss and provides more information for genetic counseling of the risk of recurrence and the management of subsequent pregnancies.

In this case report, the genetic characterization of the parents was not possible, since genetic tests are not offered free of charge in the public health system. Furthermore, we must emphasize that the mutation in the *COL1A2* gene described in this case had incomplete penetration, which causes a phenotypic variation between individuals and highlights the importance of screening the parents. On the other hand, with the sequencing of exomic regions, we missed important regions in the introns, such as microRNA regions or long noncoding RNA regions. ^21^^,^^22^

In conclusion, the diagnosis of osteogenesis imperfecta is quite complex, mainly in the prenatal stage, due to the diversity of types that exist. Therefore, the clinical application of exome sequencing during this stage is essential, since it will allow us to confirm the clinical diagnosis, determine the genetic cause, inheritance patterns, and evaluate multidisciplinary therapeutic options for adequate care, as well as carry out the correct genetic counseling. parents about the prognosis and risk of recurrence in future pregnancies. This is the first prenatal diagnosis of osteogenesis Imperfecta type II confirmed by an amniotic fluid exome sequencing study carried out in our country, thus, as a hospital, we seek to implement measures that ensure that genetic studies are carried out in pregnancies with clinical suspicion of monogenic disease.

## Consent

Written informed consent for publication of their clinical details and/or clinical images was obtained from the patient parents.

## Data Availability

All data underlying the results are available as part of the article and no additional source data are required.
