# Supplementary material for: Case Report: A prenatal diagnosis of osteogenesis imperfecta in a patient with a novel pathogenic variant in COL1A2
Source: F1000Res. 2023 Oct 30;12:603. Originally published 2023 Jun 5. [Version 3] doi: 10.12688/f1000research.131094.3 (PMC10565417; doi:10.12688/f1000research.131094.3)
Supplement: Peer review history

# Reviewer report: Reviewer response for version 3

**DOI:** 10.5256/f1000research.157884.r219332

**Published:** 2023-11-01

**Authors:** Roy Morello

**Affiliations:** 1 University of Arkansas for Medical Sciences, Little Rock, AR, USA

No further comments.

Are enough details provided of any physical examination and diagnostic tests, treatment given and outcomes?

Yes

Is the case presented with sufficient detail to be useful for other practitioners?

Yes

Is sufficient discussion included of the importance of the findings and their relevance to future understanding of disease processes, diagnosis or treatment?

Yes

Is the background of the case’s history and progression described in sufficient detail?

Yes

Reviewer Expertise:

Connective tissue diseases

I confirm that I have read this submission and believe that I have an appropriate level of expertise to confirm that it is of an acceptable scientific standard.

# Reviewer report: Reviewer response for version 2

**DOI:** 10.5256/f1000research.157046.r213068

**Published:** 2023-10-10

**Authors:** Roy Morello

**Affiliations:** 1 University of Arkansas for Medical Sciences, Little Rock, AR, USA

Most of my comments were addressed however, language issues in the Abstract have remained unchanged. See if you would like to edit these or not.

Are enough details provided of any physical examination and diagnostic tests, treatment given and outcomes?

Yes

Is the case presented with sufficient detail to be useful for other practitioners?

Yes

Is sufficient discussion included of the importance of the findings and their relevance to future understanding of disease processes, diagnosis or treatment?

Yes

Is the background of the case’s history and progression described in sufficient detail?

Yes

Reviewer Expertise:

Connective tissue diseases

I confirm that I have read this submission and believe that I have an appropriate level of expertise to confirm that it is of an acceptable scientific standard.

# Reviewer report: Reviewer response for version 1

**DOI:** 10.5256/f1000research.143903.r201010

**Published:** 2023-09-22

**Authors:** Roy Morello

**Affiliations:** 1 University of Arkansas for Medical Sciences, Little Rock, AR, USA

The authors report on a case of osteogenesis imperfecta type II that was identified in utero, showing multiple abnormalities by ultrasound, and then molecularly diagnosed by exome sequencing using DNA obtained by amniocentesis. They identified a new heterozygous Gly538Cys substitution in the *COL1A2* which is predicted to be pathogenic by multiple computational programs.

 I have the following comments/suggestions:

1. Abstract: “ *…a 27-year-old women…*” should read woman.
2. Abstract: “ *..a prenatal diagnosis of osteogenesis imperfecta was performed.*” Probably better to say ‘issued or requested or suspected’.
3. Abstract: “ *…detected by exomic sequencing in amniotic fluid CELLS*”
4. Figure 1: what is the meaning of 26 weeks next to the proband? Arrows should be singular (there is only 1 arrow)
5. “ *…drug treatment with furosemide and captopril at low doses were administrated…*”, please change to administered.
6. Discussion: “ *Currently, IAO is part of the 25 ED groups….*”. Please define what IAO stands for.
7. Discussion: “ *..but the use of exome sequencing should be carefully considered. case selection to maximize clinical utility*.” Broken sentence, please fix.
8. Discussion: “ *Furthermore, we must emphasize that the mutation in the COL1A2 gene described in this case had incomplete penetration, which causes a phenotypic variation between individuals and highlights the importance of screening the parents. On the other hand, with the sequencing of exomic regions, we missed important regions in the introns, such as microRNA regions or long noncoding RNA regions.*” Please provide some more explanation and/or context about these two sentences which are not easy to follow.
9. Discussion: “ *..the correct genetic counseling. parents about the prognosis*”. There is a period in the middle of the sentence. Please fix.

Are enough details provided of any physical examination and diagnostic tests, treatment given and outcomes?

Yes

Is the case presented with sufficient detail to be useful for other practitioners?

Yes

Is sufficient discussion included of the importance of the findings and their relevance to future understanding of disease processes, diagnosis or treatment?

Yes

Is the background of the case’s history and progression described in sufficient detail?

Yes

Reviewer Expertise:

Connective tissue diseases

I confirm that I have read this submission and believe that I have an appropriate level of expertise to confirm that it is of an acceptable scientific standard, however I have significant reservations, as outlined above.

# Reviewer report: Reviewer response for version 1

**DOI:** 10.5256/f1000research.143903.r195013

**Published:** 2023-09-21

**Authors:** Xiuli Zhao

**Affiliations:** 1 Medical Genetics, Institute of Basic Medical Sciences, Chinese Academy of Medical Sciences & School of Basic Medicine, Peking Union Medical College, Beijing, China

This paper performed a prenatal diagnosis on a fetus suspected of type II Osteogenesis Imperfecta (OI), a rare hereditary bone disease. It provides a detailed background of the case’s history and progression, including the case’s examination, diagnosis, treatment plan, and final outcomes of the fetus.

 The genotype-phenotype correlation in OI and similar diseases is still under-researched, and there is significant heterogeneity in patient phenotypes, with a persistent risk of lethal OI in fetuses. The most effective approach currently is prenatal screening for OI-related gene mutations in combination with a pregnancy diagnosis.

 The potentially pathogenic mutation *COL1A2*: c.1612G>T (p. Gly538Cys) identified in this paper is reported for the first time, expanding the spectrum of OI pathogenic mutations. Unfortunately, no genetic mutation testing or co-segregation analysis was performed on family members, which are crucial for assessing the pathogenicity of the mutation. In summary, this report presents a very typical case of lethal Type II OI, providing an important diagnostic treatment case and a significant pathogenic mutation.

Are enough details provided of any physical examination and diagnostic tests, treatment given and outcomes?

Yes

Is the case presented with sufficient detail to be useful for other practitioners?

Yes

Is sufficient discussion included of the importance of the findings and their relevance to future understanding of disease processes, diagnosis or treatment?

Yes

Is the background of the case’s history and progression described in sufficient detail?

Yes

Reviewer Expertise:

In the recent ten years, my research focus on the precise medicine of monogenic diseases, especially osteogenesis imperfecta. We perform the genetic research in Chinese population with osteogenesis imperfecta.

I confirm that I have read this submission and believe that I have an appropriate level of expertise to confirm that it is of an acceptable scientific standard.
